# (NZ)CH...O Contacts assist crystallization of a ParB-like nuclease

**DOI:** 10.1186/1472-6807-7-46

**Published:** 2007-07-07

**Authors:** Neil Shaw, Chongyun Cheng, Wolfram Tempel, Jessie Chang, Joseph Ng, Xin-Yu Wang, Sarah Perrett, John Rose, Zihe Rao, Bi-Cheng Wang, Zhi-Jie Liu

**Affiliations:** 1 National Laboratory of Biomacromolecules, Institute of Biophysics, Chinese Academy of Sciences, Beijing, 100101, China; 2 Southeast Collaboratory for Structural Genomics, Department of Biochemistry and Molecular Biology, University of Georgia, GA 30602, USA; 3 Laboratory of Structural Biology and Department of Biological Sciences, University of Alabama in Huntsville, Huntsville, AL 35899, USA; 4 Laboratory of Structural Biology, Tsinghua University, 100084, China

## Abstract

**Background:**

The major bottleneck for determination of 3 D structures of proteins using X-rays is the production of diffraction quality crystals. Often proteins are subjected to chemical modification to improve the chances of crystallization

**Results:**

Here, we report the successful crystallization of a nuclease employing a reductive methylation protocol. The key to crystallization was the successful introduction of 44 new cohesive (NZ) CH...O contacts (3.2 – 3.7 Å) by the addition of 2 methyl groups to the side chain amine nitrogen (NZ) of 9 lysine residues of the nuclease. The new contacts dramatically altered the crystallization properties of the protein, resulting in crystals that diffracted to 1.2 Å resolution. Analytical ultracentrifugation analysis and thermodynamics results revealed a more compact protein structure with better solvent exclusion of buried Trp residues in the folded state of the methylated protein, assisting crystallization.

**Conclusion:**

In this study, introduction of novel cohesive (NZ)CH...O contacts by reductive methylation resulted in the crystallization of a protein that had previously resisted crystallization in spite of extensive purification and crystallization space screening. Introduction of (NZ)CH...O contacts could provide a solution to crystallization problems for a broad range of protein targets.

## Background

The resolution and accuracy of the structural information provided by X-rays is unsurpassed when compared to other techniques employed to resolve the 3 D structure of proteins [1]. However, not all proteins can be made available for X-ray diffraction studies because of the inherent difficulty in obtaining single crystals of adequate size and quality [2]. A subset of such proteins that resist crystallization can be salvaged by employing a reductive methylation protocol [3-11]. A 26 kD nuclease from *Pyrococcus furiosus *used in the current study had resisted crystallization inspite of extensive purification and crystallization space screening. A number of techniques have been developed to improve a given target's potential for crystallization. Truncation of disordered regions [12,13], mutagenesis of surface residues [14-16] and chemical modification of proteins [3] have been proven effective in this regard and a number of protein structures have been solved successfully. We decided to modify the surface lysines of the nuclease by reductive methylation in an attempt to crystallize the protein. The rationale behind targeting the amine nitrogen (NZ) of the nuclease arose from a number of considerations. Lysines harbouring free NZ atoms almost always reside on the surface of protein molecules [17]. The thermodynamic cost for ordering the highly flexible solvent exposed side chains of lysines is exorbitant [18]. Since crystallization is a surface phenomenon, a disordered lysine side chain has a profound negative impact on the formation of stable, uniform, inter molecular contacts essential for packaging of protein molecules in a crystal lattice. Interestingly, we found that methylation of surface lysines resulted in a decrease in the free energy of folding of the protein. This is consistent with the entropic cost of reduced flexibility in the native state due to formation of new intra molecular interactions, which in turn will lower the barrier to crystallization. We further probed the effect of methylation at the molecular level and show that cohesive (NZ)CH...O bonds assisted crystallization of the nuclease.

## Results

### Generation of intra molecular contacts

**Figure 1:**
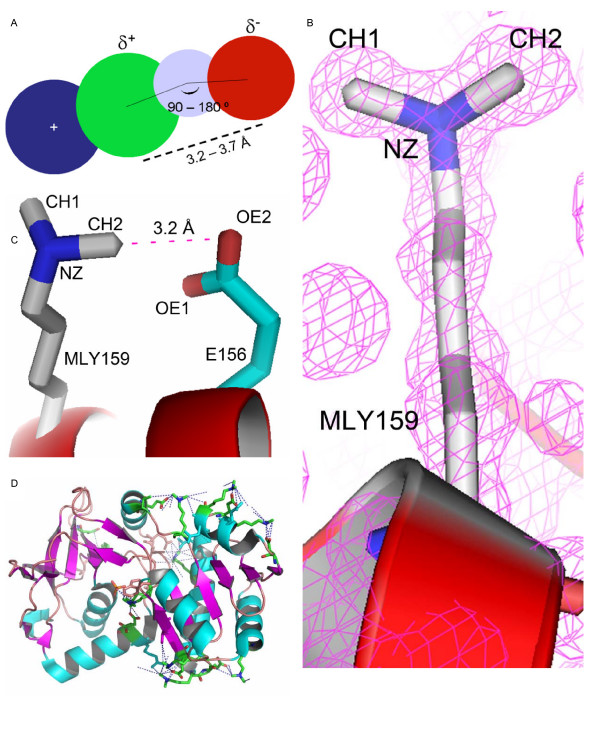
**Nature of (NZ)CH...O contacts of the methylated nuclease**. **A**. Diagrammatic representation of the (NZ)CH...O bond. The side chain amine nitrogen (NZ) of lysine residues (blue circle) polarizes the covalently linked methyl carbon (green circle). The polarized methyl carbon acts like a proton donor and forms ionic interactions with neighbouring carboxyl oxygens (red circle). The optimal range for the (NZ)CH...O bond distance is between 3.2 – 3.7 Å. The angle of the approach of a proton towards the lone pair of electrons is generally between 90 – 180°. In order to calculate the angle, the position of hydrogen (grey circle) for X-ray structures is usually deduced. **B**. Electron density for the dimethylated lysine MLY159. The 2 | Fo | – | Fc | electron density map was contoured at 1.5 σ. **C**. The protonated methyl carbon, CH2, of MLY159 is seen forming a 3.6 Å (NZ)CH...O bond with the carboxyl oxygen of E156. The amino acids are represented as sticks. The (NZ)CH...O bond is shown as a dashed magenta line. **D**. A cartoon representation of the modified protein showing the dimethylated lysines engaged in the formation of numerous intra molecular (NZ)CH...O contacts. Such contacts localize side chains and loops in space, resulting in a compact protein molecule. (NZ)CH...O bonds are shown as dashed blue lines.

After purifying the protein to homogeneity, two methyl groups were covalently linked to the free amine nitrogen of lysine residues [3-11] (Figure 1A) by treating the protein with formaldehyde and dimethylamine borane complex (DMAB). Electron density for only 9 amine nitrogens out of 32 (including the N-terminal amine group), could be unambiguously assigned as dimethylated (Figure 1B).

**Figure 2:**
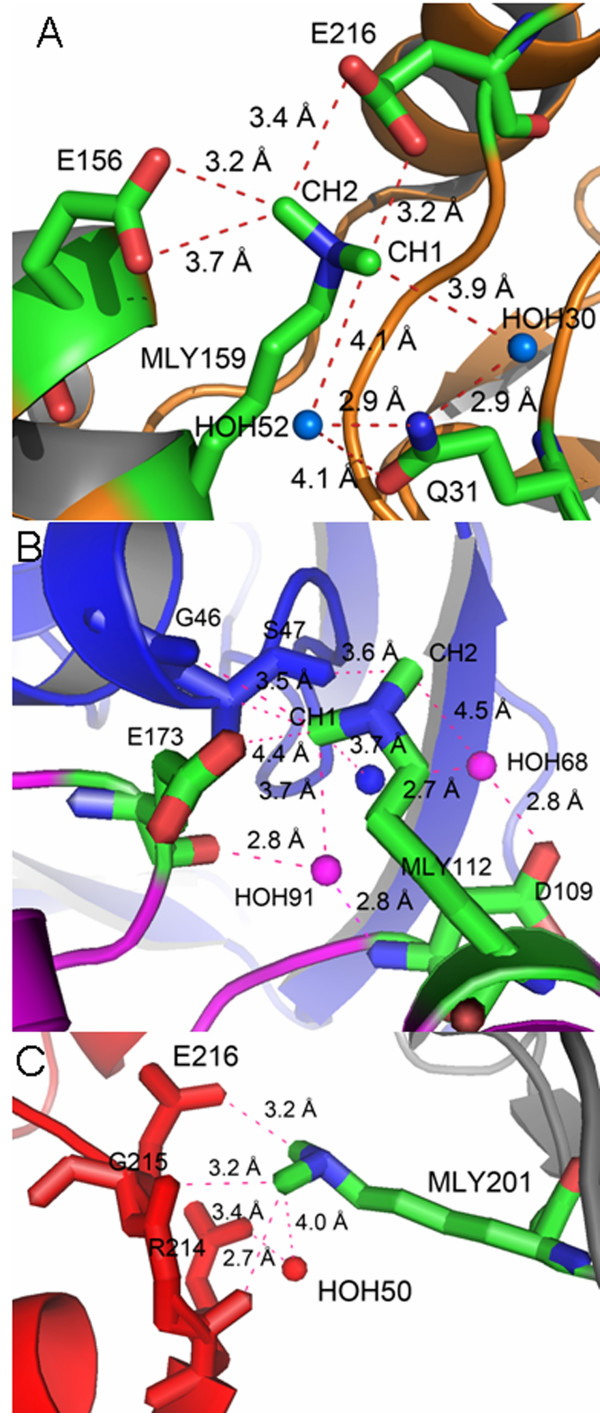
**Generation of intra and inter molecular (NZ)CH...O contacts for the nuclease**. **A**. The intra molecular (NZ)CHO contacts generated for MLY159 as a result of the chemical modification of the nuclease are shown as dashed magenta lines. **B**, **C**. The symmetry generated inter molecular contacts synthesized for MLY112 and MLY201 during the crystallization of the nuclease are shown as magenta dashed lines. Residues involved in the formation of (NZ)CH...O bonds are represented as sticks while water molecules are represented as spheres.

The methyl carbon is protonated because of the strong electron withdrawing nature of the NZ atom. The protonated methyl carbon can form ionic interactions with carbonyl and carboxyl oxygens of surrounding residues. The (NZ)CH...O bond formed by the CH2 group of MLY159 and the carboxyl oxygen OE2 of Glu156 is shown in Figure 1C. The length of the cohesive interaction is 3.27 Å. Forty new intra molecular (NZ)CH...O contacts in the range 3.2 to 4.0 Å were generated because of the methylation, of which 25 interactions were between 3.2 to 3.8 Å (see Additional file 1). The large number of cohesive intra molecular contacts generated helps immobilize the flexible regions of the protein molecules (Figure 1D), which is crucial for the formation of stable intermolecular contacts and may lower the entropic cost of crystallization. The temperature factor of the MLY159NZ was 12.18. The addition of methyl groups to the amine nitrogen and the formation of cohesive bonds through the protonated carbon of the methyl group seemed to have significantly lowered the B factor of the NZ atom of MLY159 indicating a localized side chain. Glu216 forms two strong (NZ)CH...O cohesive bonds with the methyl groups of MLY159 (Figure 2A). The B factors of the OE1 and OE2 atoms of Glu216 involved in the formation of the hydrogen bond were 14.3 and 12.36 respectively. The low B factors demonstrate immobilization of the Glu216 side chain possibly because of the new (NZ)CH...O bonds. Similarly, the B factors of both the carboxyl oxygens of Glu156 participating in (NZ)CH...O interactions were also low. B factors of 16.64 for the OE1 oxygen and 20.24 for the OE2 oxygen indicate the side chain of Glu156 to be severely restricted in movement. The B factors, however, need to be interpreted cautiously in absence of a structure of the unmodified protein for comparison.

### Generation of inter molecular contacts

The methyl carbons of all the dimethylated lysine residues were involved in the formation of multiple new symmetry-generated intermolecular contacts. The synthesis of the intermolecular contacts was initiated by gradual evaporation of the solvent in presence of different chemicals. Commercially available sparse matrix screens under oil were used for screening the best chemical environment for formation of intermolecular bonds [19,20]. The structure reveals 96 new symmetry-generated inter molecular contacts in the range 3.2 – 5.0 Å involving the (NZ)CH group and 28 of these inter molecular contacts are of the (NZ)CH...O type and in the range 3.2 – 4.0 Å (see Additional file 2). A significant number of these interactions were within the optimal range of 3.2 – 3.7 Å for CH...O hydrogen bonds [21]. The intermolecular contacts involving the methyl groups of MLY112 and MLY201 are shown in Figures 2B and 2C respectively. The B factors for the NZ atoms of MLY112 and MLY201 were 25 and 15 respectively. The new intra and inter molecular contacts formed by the covalently linked methyl groups with the surrounding residues seem to have lowered the B factor values of the NZ atom indicating a localized side chain. This helps the packaging of the molecules in the crystal and the formation of a compact crystalline lattice.

**Figure 3:**
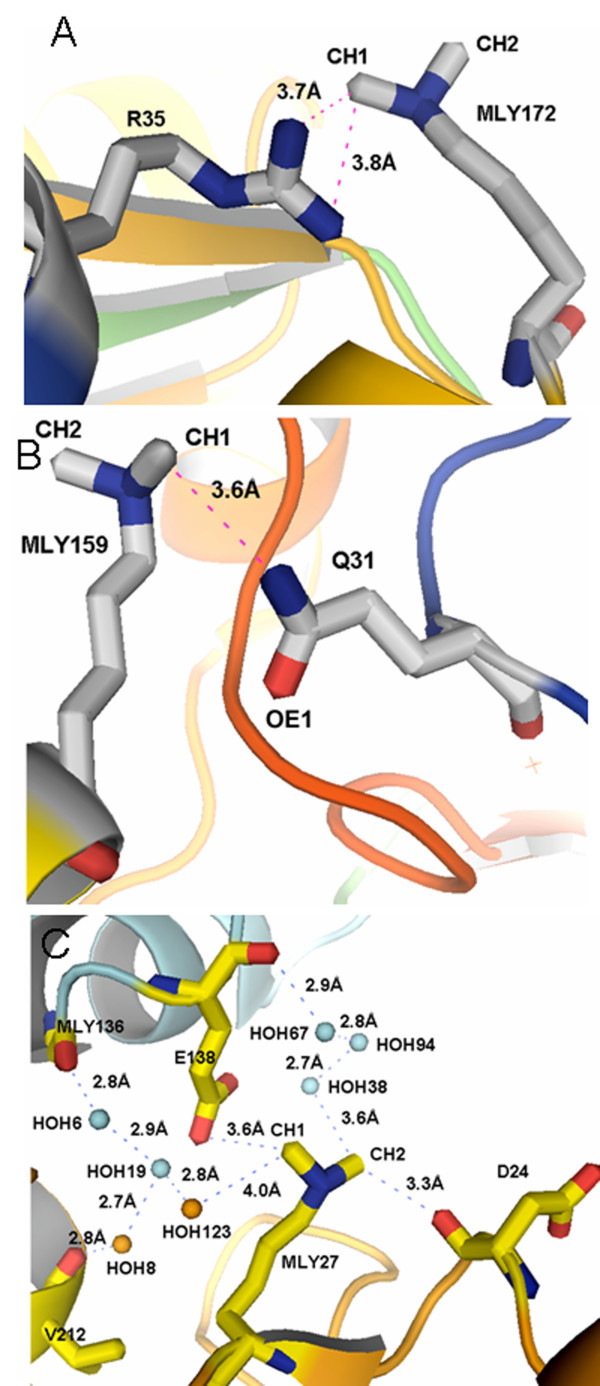
**(NZ)CHN contacts and water molecules in (NZ)CHO bonds observed for the methylated nuclease**. **A, B**. The side chain nitrogens of R35 and Q31 are seen forming (NZ)CHN bonds with the methyl groups of MLY172 and MLY159 respectively. Excess positive charge on the nitrogen is dispersed via ionic interactions mediated by water molecules [22] or as in case of Q31, relayed directly to electron negative oxygen OE1. **C**. A number of water molecules are seen participating in the formation of (NZ)CHO bonds. Residues involved in the formation of (NZ)CHN and (NZ)CH...O bonds are represented as sticks while water molecules are represented as spheres.

Although the contacts formed were predominantly (NZ)CH...O bonds, a few (NZ)CH...N bonds were also formed (Figure 3A and 3B). The side chain nitrogens of Arg35 interact with the CH1 methyl carbon of MLY172. Water molecules surrounding these interactions possibly help disperse the excess positive charge on the nitrogens by bridging ionic interactions [22]. The NH1 nitrogen of Arg35 forms a hydrogen bond with water 119, which is hydrogen bonded to the carboxylic oxygens, OD1 and OD2, of Asp 39. Similarly, the NH2 nitrogen of Arg35 forms a hydrogen bond with water 117, which is hydrogen bonded to the carbonyl oxygen of Gln170. The excess positive charge on the Arg35 nitrogens is relayed to the carboxyl oxygens of Asp35 and carbonyl oxygen of Gln170 via water molecules (data not shown). Similarly, in case of Gln31, the excess positive charge is relayed to the side chain OE1 oxygen atom.

In addition, a significant number of water molecules were observed to form (NZ)CH...O contacts (Figure 3C).

### Analytical ultracentrifugation

**Figure 4:**
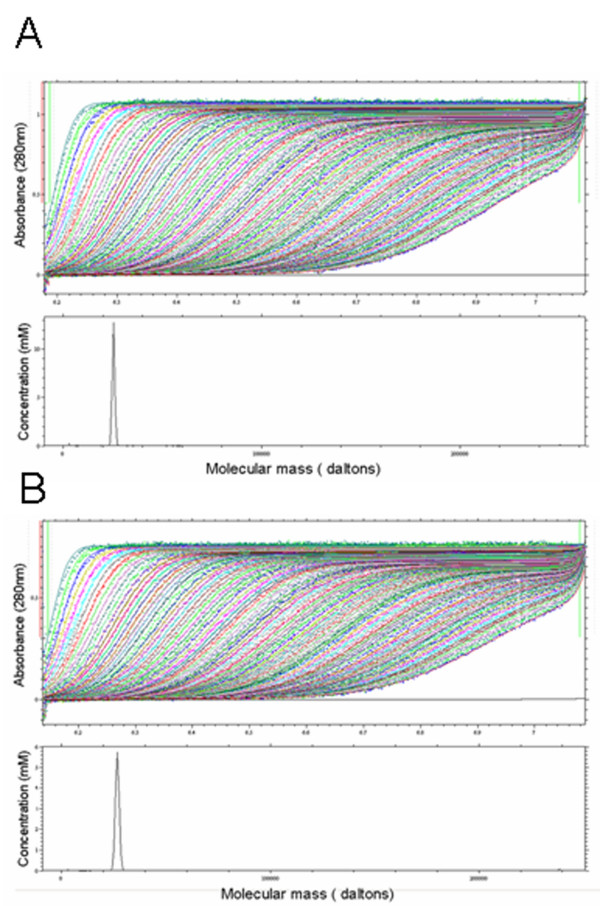
**Analytical ultracentrifugation analysis of methylated and non-methylated protein**. The methylated **(A) **and non-methylated **(B) **protein were subjected to analytical ultracentrifugation analysis as described in Methods. The sedimentation velocity experiments showed the protein remained pure and monomeric after the chemical modification. There was an obvious decrease in the diffusion co-efficient for the protein after the methylation indicating a more compact shape.

The 26 kD protein was subjected to analytical ultracentrifugation analysis in order to determine the effect of the reductive methylation protocol on the purity, aggregation state and shape of the protein. The sedimentation velocity experiment results revealed that both non-methylated and methylated proteins were pure, homogenous and monomeric (Figure 4). However, a qualitative decrease in the diffusion co-efficient (D) could be observed for the methylated protein (Figure 4B). This is consistent with decreased flexibility of side chains and a more compact structure in the methylated protein.

### Thermodynamic stability of nuclease before and after modification

**Figure 5:**
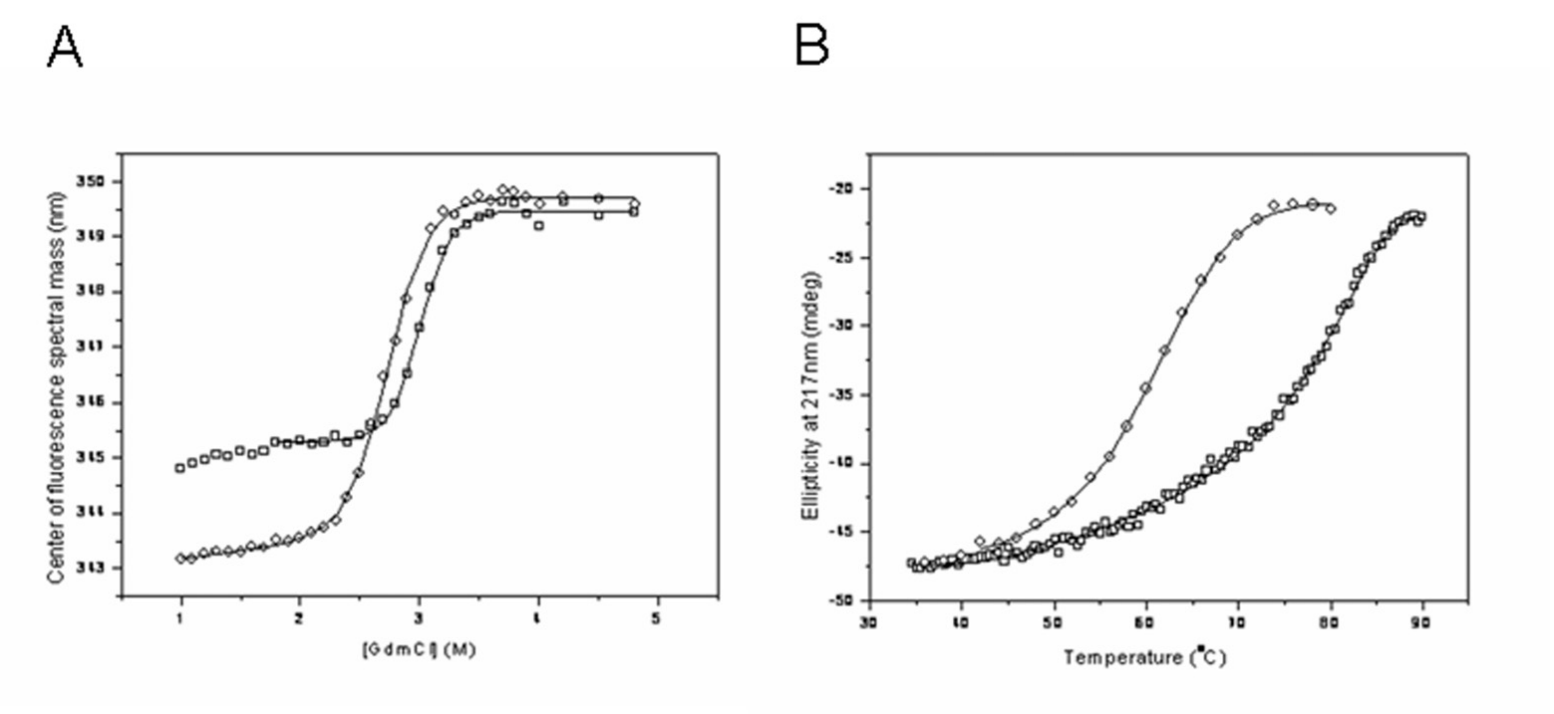
**Comparison of thermodynamic stability of modified and non-modified protein**. **A**. GdmCl-induced equilibrium denaturation of the nuclease (squares) and its modified variant (circles) in 20 mM phosphate buffer pH 7.4 at 25°C. The protein concentration was 1 μM. The fit to a two-state model is shown and the parameters obtained are displayed in Table 1. **B**. Thermally-induced equilibrium denaturation of the nuclease (squares) and its modified variant (circles) in 20 mM phosphate buffer pH 7.4 in the presence of 2.5 M GdmCl. The protein concentration was 25 μM. The fit to a two-state model is shown and the free energy value obtained after extrapolation to standard conditions (25°C in the absence of GdmCl) is the same within error as that obtained by GdmCl denaturation (Table 1).

In order to further investigate the mechanism by which chemical modification of the protein affected the ease of crystallization, we performed equilibrium denaturation experiments using heat or the chaotropic agent guanidinium chloride (GdmCl) as denaturants. The nuclease contains three Trp residues, of which two (W74 and W102) are buried in the folded structure, making intrinsic fluorescence a sensitive probe of global structural changes. We also used far-UV circular dichroism (CD), which monitors changes in secondary structure. Both proteins were found to be extremely thermostable and no secondary structural changes were detected below 90°C (not shown). However, we found that the proteins could be completely unfolded in 4 M GdmCl at 25°C (Figure 5A). Further, thermal denaturation could be achieved in the presence of a non-denaturing concentration of GdmCl (Figure 5B). Interestingly, we found that the chemical modification resulted in a decrease in the mid-point of unfolding in both GdmCl and thermal denaturation (Figures 5A, B), which corresponds to a decrease in the free energy of unfolding, Δ *G*_U,H20_, of 18 ± 3 kJ/mol (Table 1). Surface lysines are likely to be involved in salt-bridges or other favourable interactions that may be lost on methylation, resulting in a decrease in stability. Another explanation is that there is a decrease in entropy in the folded state of the modified protein due to formation of intra molecular (NZ)CH...O interactions involving the introduced methyl groups, which reduces the flexibility of the protein. Solvent effects may also contribute to the observed difference in stability, for example, greater ordering of water molecules around hydrophobic methyl groups at the protein surface could also lead to a greater loss in entropy on folding for the methylated protein. We also observed that the λ_max _of the fluorescence spectrum for the native state was blue-shifted for the modified protein, whereas the λ_max _for the denatured states was similar (Figure 5A). This is consistent with a more compact structure and better solvent exclusion of buried Trp residues in the folded state of the chemically modified protein. Reduced entropy of the solvated folded structure, together with the formation of additional favourable interactions in the crystalline state, may account for the greater ease of crystallization after the chemical modification

**Table 1:** Comparison of thermodynamic parameters for ParB before and after modification^§^

Protein	[GdmCl]_1/2_	*m*	ΔG_U,H2O_
	(M)	(kJ/mol/M)	(kJ/mol)
Nuclease	3.01 ± 0.01	19 ± 1	57.4 ± 3.0
Nuclease (modified)	2.74 ± 0.01	14.4 ± 0.5	39.4 ± 1.4

^§ ^The values for GdmCl denaturation were obtained by fitting data to a standard 2-state model [29] where [GdmCl]_1/2 _is the midpoint and *m *the slope of the denaturation transition. Δ*G*_U,H2O _is the free energy of unfolding at 25°C in the absence of denaturant. The errors shown are the standard errors from the fit. The dependence of Δ*G*_U _on the GdmCl concentration is given by Δ*G*_(GdmCl) _= Δ G_u_-*m *[GdmCl]. Conditions were as described in the legend for Figure 5A. The thermal denaturation data (Figure 5B) was also fitted to a 2-state model and was extrapolation to standard conditions (25°C in the absence of denaturant) as described previously [30]. The value of Δ*G*_U,H2O _thus obtained is the same within the error as that shown above.

## Discussion

Structure determination holds the key to unravelling the mechanisms by which proteins drive the machinery of all living organisms for survival and propagation. In spite of several dramatic technological advances in automation and information science, the key step for successful crystallographic structure determination – production of high quality crystals – continues to remain a resource intensive bottleneck [1]. A first step towards addressing this bottleneck would be to identify the variables involved in crystallization. The nature of the protein is the single most important factor that influences crystallization. From a crystallization point of view – size, charge, hydrophobicity, hydrophilicity, flexibility, oxidation state and post-translational modifications like phosphorylation, glycosylation and myristylation – define the nature of a protein. Since the nature of the protein often varies with function, there is no universal crystallization strategy that would work for all proteins. To maximize the chances of crystallization it is crucial to identify and target an attribute of the protein that would have a profound effect on the crystallization. Surface lysines offer one such target. Lysines almost always reside on the surface of proteins. The solvent exposed side chains of lysines are highly mobile and prevent the formation of inter molecular contacts essential for the assembly of a crystalline lattice [18]. Defects observed in protein crystals like low resolution and twinning is also a manifestation of the flexibility of the side chains found on the surface of the protein.

Locking the side chain amine nitrogen of lysines with the electron negative carboxyl oxygens of glutamic and aspartic acid side chains via cohesive ionic interactions will result in the immobilization of these side chains (Figure 2). Although the presence of CH...O bonds has been demonstrated in proteins, nucleic acids and carbohydrates [21-27], the (NZ)CH...O bonds introduced in the current study have never been described before. Methyl groups are very effective in mediating and bridging the physical distances between the free amine nitrogen and the side chain carboxyl oxygens for the formation of cohesive ionic interactions. Methyl groups can be covalently linked to the free amine nitrogen (NZ) with very high specificity using formaldehyde as the methyl group donor in the presence of dimethylamine borane complex [3]. The amine nitrogen sitting adjacent to the methyl carbon, (NZ)CH, has an inductive effect on the methyl carbon resulting in a highly polarized carbon, which can act as a proton donor. The negative charge required for the electro neutrality is compensated by the lone pair of electrons of carboxyl oxygens found on the side chains of neighbouring glutamic and aspartic acid residues. The net effect is a cohesive ionic (NZ)CH...O interaction between the side chains leading to a compact, rigid protein molecule with localized side chains and loops. The positive charge on the methyl carbon can also be dispersed indirectly via the participation of water molecules (Figure 3C).

**Figure 6:**
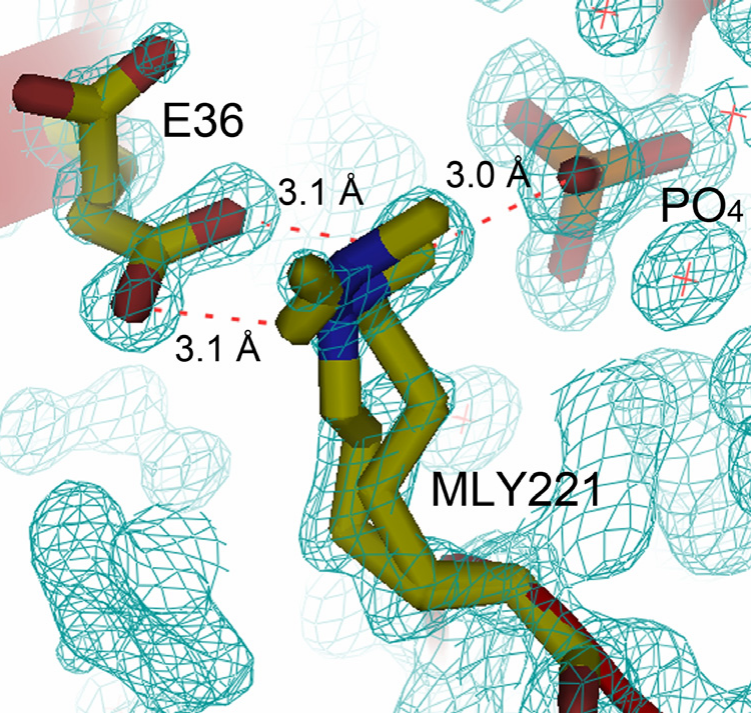
**Occupancy of MLY221**. MLY221 shows 2 conformations. Conformation A has occupancy of 65%. The distance of the oxygen atoms of E36 and the PO^- ^_4 _ligand from the (NZ)CH group of the MLY221 is shorter than the permissible limit of 3.2 Å for (NZ)CHO bonds, resulting in the distorting of MLY221 and E36. The 2 | Fo | – | Fc | electron density map was contoured at 1.5 σ

The maximum and minimum distances for a cohesive CH...O bond have been set at 3.2 Å and 3.7 Å respectively, depending on the direction of the approach of the proton towards the lone pair of electrons of oxygen [21]. Deviations in the above distances are frequently observed owing to the steric interactions between the atoms involved in the formation of the (NZ)CH...O bonds and the surrounding residues. In the present study, except for a couple of contacts involving MLY221, the minimum distance of all the CH...O bonds was 3.2 Å. The only short contacts – a 3.0 Å link between the CH2 methyl carbon of MLY221 and a phosphate oxygen, and a 3.1 Å bond between the carboxyl oxygens of E36 and the MLY221 methyl carbons, resulted in the distortion of the conformation of the MLY221 and the glutamic acid (Figure 6), suggesting steric clashes due to the excessive closeness of the methyl carbon and the oxygen atoms. Thus, 3.2 Å seems to be the minimum van der Waals limit for all (NZ)CH...O interactions. Detailed analysis of the electron density map showed no other obvious changes to any other amino acid confirming the specificity of the modification.

The methylated protein lost its ability to cleave DNA (results not shown). The dimethylation of K221 sitting at the edge of the active site may sterically hinder access of the active site to the incoming DNA. It is also possible that an overall conformational change in the protein induced by the chemical modification affects the catalytic site and compromises the function of the protein. However, such loss-of-function due to methylation has not been reported previously. Further studies are warranted in order to determine the exact cause for the inactivation of the methylated protein.

When a protein is set up for crystallization, the molecules are moving randomly in search of compatible bonding partners. Some of the inter molecular contacts generated during the course of random collisions are sustained. As more and more solvent evaporates, a number of these interactions become permanent. Eventually it leads to one of two possible outcomes. If the inter molecular bonding is heterogeneous, as in case of protein molecules that assume more than one conformation due to the presence of unstructured domains, flexible loops and side chains, or presence of partially unfolded regions, this will result in a disordered protein aggregate commonly referred to as precipitate. Poorly diffracting or defective crystals are also a consequence of the conformational flexibility of protein molecules. A homogenous inter molecular bonding pattern between protein molecules, as in the case of structurally rigid molecules, results in optimal packing of the molecules in a crystal lattice. A direct manifestation of the chemical modification of the protein is the reduction in number of degrees of freedom available to the protein for assuming different conformations. Introduction of (NZ)CH...O bonds curbs the movement of side chains and fixes their orientation in space. This produces uniform bonding partners and decreases the steric clashes between molecules during the packing of the lattice. The observed reduction in the free energy of folding for the modified protein is consistent with reduced flexibility leading to lower entropy in the native state; ordering of water molecules around the surface-exposed methyl groups may also contribute to this. Crystallization will be favoured for the modified protein by the reduction in entropy of the solvated structure and the involvement of the methyl groups in inter molecular (NZ)CH...O interactions upon crystallization

**Figure 7:**
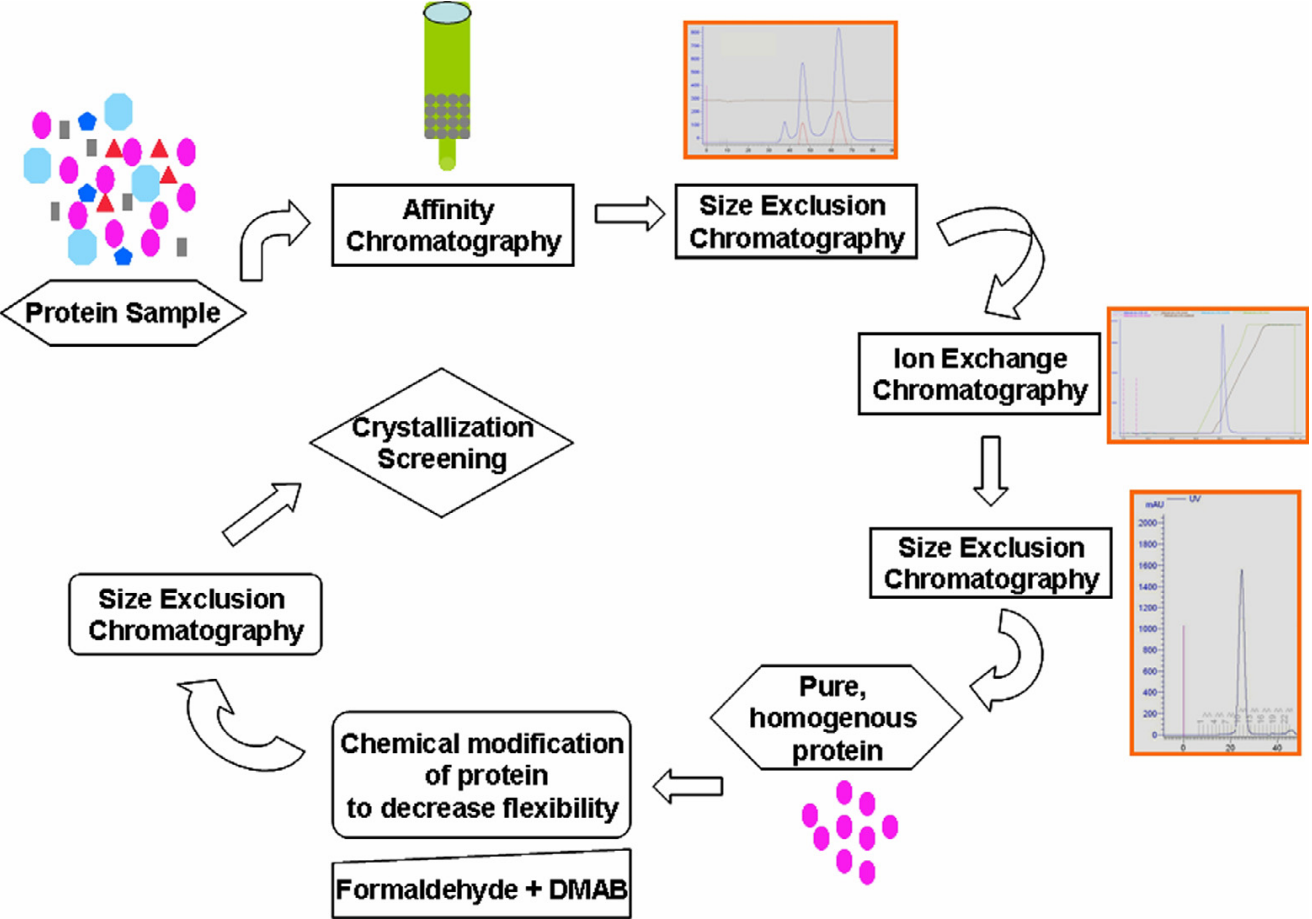
**Process flow sheet of the crystallization strategy for the nuclease**. The protein sample is purified to homogeneity using a combination of chromatographic methods. The pure and homogenous protein is chemically modified in order to localize the side chains and loops. The resultant compact protein molecule is then screened against a variety of chemical environments using commercially available sparse matrix screens to determine the best condition for the self-assembly of the protein molecules into a crystalline lattice.

A pre-requisite to the success of the crystallization strategy described here is the requirement of a highly pure homogenous protein sample [28] usually obtained by a combination of different chromatography steps (Figure 7). Presence of homologous and heterologous impurities can compromise the effectiveness of the chemical modification of the protein.

## Conclusion

In conclusion, introduction of (NZ)CH...O bonds by reductive methylation of surface lysines as a means to salvage targets is simple, fast, economical and non laborious. It could be the first method of choice for rescuing a target before attempting a more extensive approach involving mutagenesis.

## Methods

### Protein production and purification

The 26 kD ParB nuclease was expressed with a N-terminal hexa Histidine tag. The gene was PCR amplified from the genomic DNA of *Pyrococcus furiosus *and cloned into pET-28a vector (Invitrogen). *E coli BL21 *cells containing the plasmid were grown in LB cultures. The protein was purified using affinity chromatography followed by size exclusion chromatography. Nucleic acids were removed by hydroxyapatite chromatography (GE Healthcare). Further purification was achieved by an ion exchange step. The protein was exchanged into crystallization buffer (20 mM Tris, pH8.0, 200 mM NaCl) using a size exclusion column.

### Chemical modification

Methylation of the protein was done as described before [3] using formaldehyde and dimethylamine-borane complex (DMAB). In brief, 10 mg/ml of protein in a 1.5 ml eppendorf tube covered with aluminium foil was mixed with 40 μl of 1 M solution of formaldehyde (Sigma) and 20 μl of 1 M solution of DMAB (Sigma) in the dark at 4°C. The reaction mixture was incubated under shaking conditions for 2 h, after which the chemical additions were repeated. Finally, 10 μl of DMAB was added and the reaction mixture was incubated overnight. Excess chemicals were removed by size exclusion chromatography.

### Crystallization

Methylated and non-methylated protein was set up for crystallization under oil as described before [19,20]. 1-μl crystallization drops contained 0.5 μl protein mixed with 0.5 μl of crystallization solution. Commercially available sparse matrix screens (Hampton Research, Molecular Dimensions) were used for crystallization screening. Crystals for structure determination were produced using a precipitant solution consisting of 600 mM sodium di-hydrogen phosphate, 2.4 M di-potassium hydrogen phosphate, 200 mM sodium chloride, 100 mM HEPES, p H 7.3.

### Data collection and structure determination

Data collection and structure determination will be described elsewhere.

### Analytical Ultracentrifugation

Analytical sedimentation velocity experiments were carried out using a ProteomeLab™ XL-I protein characterization system (Beckman Coulter). An-60Ti rotor was used to centrifuge a 10 mg/ml protein sample suspended in 50 mM phosphate buffer, 150 mM NaCl, pH 7.2, at 60,000 rpm. Absorbance was read at 280 nm. A set of 93 scans were collected at 1 min intervals. Data was analyzed using Sedfit software

### Equilibrium denaturation experiments

All fluorescence denaturation experiments were performed in 20 mM phosphate buffer pH 7.4 at 25°C, with a final protein concentration of 1.2 μM. Samples of modified and unmodified protein were mixed with different concentrations of GdmCl and allowed to equilibrate overnight before measurements were taken. Refolding experiments were also performed, by denaturing the protein for 8 h in 6 M GdmCl, then diluting the protein to give different final concentrations of GdmCl as for unfolding experiments. The intrinsic fluorescence spectra were recorded between 300 and 400 nm after excitation at 280 nm in a Hitachi F-4500 spectrofluorimeter. The fluorescence data were plotted as the centre of spectral mass as described previously [29]. GdmCl denaturation was found to be reversible and the data were fitted to a 2-state model [30].

Far-UV CD experiments were performed on a Pi-star 180 instrument (Applied Photophysics, UK) using a cell of 1 mm optical path length and the same buffer as for fluorescence experiments. The protein concentration was 25 μM. The temperature was changed at a rate of 1°C per 10 min, with a step size of 0.5°C, for both heating and cooling. Thermal denaturation was found to be reversible and the data were analyzed as described previously [31]

## List of abbreviations used

MLY – Methylated lysine

(NZ) – Side chain amine nitrogen

## Authors' contributions

NS, JC, and JN produced, purified, and crystallized the protein. NS and CC did the chemical modification and analytical ultracentrifugation analysis. X-YW and SP carried out the thermodynamic characterization of the protein. Z-JL, WT, and JR solved the structure and did the analysis. Z-JL designed the experiments and interpreted the data. B-CW and ZR conceived the study and participated in its design and co-ordination. NS, SP, and Z-JL drafted the manuscript. All authors read and approved the final manuscript.

## Accession numbers

Coordinates for the structure reported in the current study have been deposited in the Protein Data Bank under accession code 1VK1

## Supplementary Material

Additional file 1 **Intra molecular contacts$ of the (NZ)CH group of methylated lysines of the nuclease**. The data presented in the table is a list of intra molecular contacts generated during the methylation of the nuclease. Click here for file

Additional file 2 Symmetry generated contacts* of the (NZ)CH group of the methylated lysines of the nuclease. The data presented in the table is a list of intra molecular contacts generated during the methylation of the nuclease. Click here for file
