# Supplementary material for: (NZ)CH...O Contacts assist crystallization of a ParB-like nuclease
Source: BMC Struct Biol. 2007 Jul 7;7:46. doi: 10.1186/1472-6807-7-46 (PMC1940005; doi:10.1186/1472-6807-7-46)
Supplement: Additional file 1 — Intra molecular contacts$ of the (NZ)CH group of methylated lysines of the nuclease. The data presented in the table is a list of intra molecular contacts generated during the methylation of the nuclease. [file 1472-6807-7-46-S1.doc]

# Additional files

### Additional file 1

### File format: DOC

### Title: Intra molecular contacts^$^ of the (NZ)CH group of methylated lysines of the nuclease

Description: The data presented in the table is a list of intra molecular contacts generated during the methylation of the nuclease.

­­­­­­­­­­­­­­­­­­­­­­­­­­­­­­­­­­­­­­­­­­­­­­­­­­­­­­­

Source Atoms Target Atoms Distance

(Å)

MLY27CH1 71HOH 3.7

123HOH 4.2

MLY27CH2 24AspO 3.3

64GluOE2 3.6

MLY107CH1 177ValCG1 4.8

MLY107CH2 177ValCG1 4.7

175GluOE2 4.7

MLY112CH1 68HOH 4.5

MLY112CH2 91HOH 3.8

173GluO 4.1

173GluOE2 4.5

MLY133CH1 127AspOD2 3.4

38HOH 3.9

80HOH 3.9

94HOH 4.0

MLY133CH2 127AspOD1 3.8

127AspOD2 3.4

MLY136CH1 138GluOE2 3.2

19HOH 4.1

MLY136CH2 132GluOE2 3.8

44HOH 4.4

MLY159CH1 31GlnNE2 3.7

217Val) 3.9

216GluOE2 3.2

217ValCG2 3.8

52HOH 4.1

33HOH 3.8

124HOH 4.2

216GluOE1 4.4

156GluOE1 4.9

156GluOE2 5.0

MLY159CH2 216GluOE1 3.5

216GluOE2 3.3

156GluOE1 3.8

156GluOE2 3.3

217ValO 3.8

155LeuCD2 3.9

155LeuCD1 4.9

155LeuCG 4.1

33HOH 3.8

97HOH 4.6

MLY172CH1 35ArgNH1 3.7

35ArgNH2 3.8

175GluOE2 3.2

48HOH 3.7

126HOH 3.9

119HOH 4.9

MLY172CH2 175GluOE1 3.6

175GluOE2 3.4

48HOH 4.7

MLY201CH1 23HOH 3.3

136MLYO 3.6

135GluO 4.8

6HOH 4.5

MLY201CH2 23HOH 3.3

47HOH 3.3

204ThrCG2 4.5

207GluOE2 5.0

137HOH 4.3

MLY221CH1A 102TrpCE2 3.9

102TrpCZ2 3.8

102TrpCH2 3.8

36GluOE1 3.5

36GluOE2 3.5

36GluCD 3.9

MLY221CH1B 36GluOE1 3.4

36GluOE2 3.1

36GluCD 3.7

178TyrCB 3.7

MLY221CH2A 36GluOE1 3.1

301PO4O3 3.0

93HOH 3.8

45HOH 3.9

102TrpNE1 3.8

102TrpCE2 3.6

102TrpCZ2 3.3

102TrpCH2 3.9

MLY221CH2B 36GluOE1 3.8

301PO4O3 3.4

93HOH 3.8

^$^ The intra molecular contacts were calculated using program CONTACT of CCP4 suite [32]. MLY221 showed 2 conformations, which are represented as A and B.
