# Supplementary material for: (NZ)CH...O Contacts assist crystallization of a ParB-like nuclease
Source: BMC Struct Biol. 2007 Jul 7;7:46. doi: 10.1186/1472-6807-7-46 (PMC1940005; doi:10.1186/1472-6807-7-46)
Supplement: Additional file 2 — Symmetry generated contacts* of the (NZ)CH group of the methylated lysines of the nuclease. The data presented in the table is a list of intra molecular contacts generated during the methylation of the nuclease. [file 1472-6807-7-46-S2.doc]

Additional file 2

File format: DOC

Title: Symmetry generated contacts* of the (NZ)CH group of the methylated lysines of the nuclease

Description: The data presented in the table is a list of intra molecular contacts generated during the methylation of the nuclease.

Source Atoms Target Atoms Distance Symmetry Operation

(Å)

MLY27CH1 138GluCD 3.68 [ +1C] 3: -X+1/2, Y+1/2, -Z

138GluOE1 4.26 [ +1C] 3: -X+1/2, Y+1/2, -Z

138GluOE2 3.60 [ +1C] 3: -X+1/2, Y+1/2, -Z

19HOH 3.98 [ +1C] 3: -X+1/2, Y+1/2, -Z

138GluCB 4.47 [ +1C] 3: -X+1/2, Y+1/2, -Z

138GluCG 3.80 [ +1C] 3: -X+1/2, Y+1/2, -Z

38HOH 4.06 [ +1C] 3: -X+1/2, Y+1/2, -Z

67HOH 3.77 [ +1C] 3: -X+1/2, Y+1/2, -Z

94HOH 4.33 [ +1C] 3: -X+1/2, Y+1/2, -Z

MLY27CH2 38HOH 3.57 [ +1C] 3: -X+1/2, Y+1/2, -Z

67HOH 4.65 [ +1C] 3: -X+1/2, Y+1/2, -Z

94HOH 3.85 [ +1C] 3: -X+1/2, Y+1/2, -Z

MLY112CH1 53HOH 4.19 [ ] 1: -X, Y, -Z

47SerC 4.54 [ ] 1: -X, Y, -Z

47SerO 3.69 [ ] 1: -X, Y, -Z

MLY112CH2 47SerCB 4.84 [ ] 1: -X, Y, -Z

53HOH 3.72 [ ] 1: -X, Y, -Z

46GlyC 4.53 [ ] 1: -X, Y, -Z

46GlyO 3.57 [ ] 1: -X, Y, -Z

47SerN 4.70 [ ] 1: -X, Y, -Z

47SerCA 3.87 [ ] 1: -X, Y, -Z

47SerC 3.95 [ ] 1: -X, Y, -Z

47SerO 3.51 [ ] 1: -X, Y, -Z

MLY133CH1 64GluOE2 4.89 [ -1B+1C] 3: -X+1/2, Y+1/2, -Z

MLY133CH2 64GluOE2 4.84 [ -1B+1C] 3: -X+1/2, Y+1/2, -Z

MLY136CH1 213LysCA 4.92 [ -1B+1C] 3: -X+1/2, Y+1/2, -Z

212ValC 4.56 [ -1B+1C] 3: -X+1/2, Y+1/2, -Z

59IleCG2 4.96 [ -1B+1C] 3: -X+1/2, Y+1/2, -Z

MLY27CB 4.59 [ -1B+1C] 3: -X+1/2, Y+1/2, -Z

59IleCD1 3.88 [ -1B+1C] 3: -X+1/2, Y+1/2, -Z

212ValO 3.58 [ -1B+1C] 3: -X+1/2, Y+1/2, -Z

212ValCG2 4.89 [ -1B+1C] 3: -X+1/2, Y+1/2, -Z

34HOH 3.57 [ -1B+1C] 3: -X+1/2, Y+1/2, -Z

MLY27CG 3.93 [ -1B+1C] 3: -X+1/2, Y+1/2, -Z

MLY27CD 4.33 [ -1B+1C] 3: -X+1/2, Y+1/2, -Z

8HOH 3.46 [ -1B+1C] 3: -X+1/2, Y+1/2, -Z

64GluCD 4.75 [ -1B+1C] 3: -X+1/2, Y+1/2, -Z

64GluOE1 3.55 [ -1B+1C] 3: -X+1/2, Y+1/2, -Z

MLY27CE 4.58 [ -1B+1C] 3: -X+1/2, Y+1/2, -Z

MLY136CH2 213LysCA 4.27 [ -1B+1C] 3: -X+1/2, Y+1/2, -Z

213LysC 4.62 [ -1B+1C] 3: -X+1/2, Y+1/2, -Z

213LysO 4.18 [ -1B+1C] 3: -X+1/2, Y+1/2, -Z

213LysCB 4.98 [ -1B+1C] 3: -X+1/2, Y+1/2, -Z

107HOH 4.22 [ -1B+1C] 3: -X+1/2, Y+1/2, -Z

59IleCG2 4.13 [ -1B+1C] 3: -X+1/2, Y+1/2, -Z

213LysCG 4.91 [ -1B+1C] 3: -X+1/2, Y+1/2, -Z

59IleCD1 4.77 [ -1B+1C] 3: -X+1/2, Y+1/2, -Z

212ValO 4.41 [ -1B+1C] 3: -X+1/2, Y+1/2, -Z

34HOH 3.27 [ -1B+1C] 3: -X+1/2, Y+1/2, -Z

62ThrCG2 4.15 [ -1B+1C] 3: -X+1/2, Y+1/2, -Z

64GluOE1 4.16 [ -1B+1C] 3: -X+1/2, Y+1/2, -Z

MLY159CH1 83HOH 4.53 [ +1C] 3: -X+1/2, Y+1/2, -Z

MLY159CH2 83HOH 3.97 [ +1C] 3: -X+1/2, Y+1/2, -Z

MLY172CH1 39AspOD1 4.90 [ ] 1: -X, Y, -Z

42GluOE1 4.85 [ ] 1: -X, Y, -Z

MLY172CH2 39AspCG 4.02 [ ] 1: -X, Y, -Z

39AspOD2 4.64 [ ] 1: -X, Y, -Z

39AspOD1 4.07 [ ] 1: -X, Y, -Z

39AspCB 3.90 [ ] 1: -X, Y, -Z

43SerOG 3.18 [ ] 1: -X, Y, -Z

127HOH 4.07 [ ] 1: -X, Y, -Z

39AspC 4.13 [ ] 1: -X, Y, -Z

39AspCA 3.96 [ ] 1: -X, Y, -Z

39AspO 3.52 [ ] 1: -X, Y, -Z

42GluCB 4.32 [ ] 1: -X, Y, -Z

42GluOE1 4.08 [ ] 1: -X, Y, -Z

43SerCB 4.49 [ ] 1: -X, Y, -Z

43Ser N 4.48 [ ] 1: -X, Y, -Z

43SerCA 4.83 [ ] 1: -X, Y, -Z

MLY201CH1 50HOH 4.03 [ -1B+1C] 3: -X+1/2, Y+1/2, -Z

216GluOE1 4.72 [ -1B+1C] 3: -X+1/2, Y+1/2, -Z

214ArgC 4.48 [ -1B+1C] 3: -X+1/2, Y+1/2, -Z

215GlyN 4.74 [ -1B+1C] 3: -X+1/2, Y+1/2, -Z

216GluN 4.21 [ -1B+1C] 3: -X+1/2, Y+1/2, -Z

216GluCA 4.53 [ -1B+1C] 3: -X+1/2, Y+1/2, -Z

216GluCB 4.78 [ -1B+1C] 3: -X+1/2, Y+1/2, -Z

214ArgO 3.47 [ -1B+1C] 3: -X+1/2, Y+1/2, -Z

215GlyCA 4.02 [ -1B+1C] 3: -X+1/2, Y+1/2, -Z

215GlyC 3.54 [ -1B+1C] 3: -X+1/2, Y+1/2, -Z

215GlyO 3.17 [ -1B+1C] 3: -X+1/2, Y+1/2, -Z

17HOH 4.30 [ -1B+1C] 3: -X+1/2, Y+1/2, -Z

HOH124 4.29 [ -1B+1C] 3: -X+1/2, Y+1/2, -Z

MLY201CH2 214ArgNH1 4.08 [ -1B+1C] 3: -X+1/2, Y+1/2, -Z

216GluCG 4.58 [ -1B+1C] 3: -X+1/2, Y+1/2, -Z

50HOH 3.46 [ -1B+1C] 3: -X+1/2, Y+1/2, -Z

216GluCD 3.99 [ -1B+1C] 3: -X+1/2, Y+1/2, -Z

216GluOE1 3.18 [ -1B+1C] 3: -X+1/2, Y+1/2, -Z

216GluOE2 4.76 [ -1B+1C] 3: -X+1/2, Y+1/2, -Z

33HOH 4.15 [ -1B+1C] 3: -X+1/2, Y+1/2, -Z

88HOH 3.76 [ -1B+1C] 3: -X+1/2, Y+1/2, -Z

97HOH 3.80 [ -1B+1C] 3: -X+1/2, Y+1/2, -Z

216GluCA 4.90 [ -1B+1C] 3: -X+1/2, Y+1/2, -Z

216GluCB 4.44 [ -1B+1C] 3: -X+1/2, Y+1/2, -Z

214ArgO 4.57 [ -1B+1C] 3: -X+1/2, Y+1/2, -Z

215GlyO 4.84 [ -1B+1C] 3: -X+1/2, Y+1/2, -Z

124HOH 4.52 [ -1B+1C] 3: -X+1/2, Y+1/2, -Z

___________________________________________________________________________________

* Symmetry generated inter molecular contacts were detected using NCONT program of CCP4 suite [32].
